# Physical Fitness, Nutrition and Quality of Life in German Medical Students

**DOI:** 10.3390/nu14245375

**Published:** 2022-12-17

**Authors:** Lukas Walnik, Momme Kück, Uwe Tegtbur, Volkhard Fischer, Arno Kerling

**Affiliations:** 1 Department of Rehabilitation and Sports Medicine, Hannover Medical School, 30625 Hannover, Germany; 2 Dean of Students Office, Hannover Medical School, 30625 Hannover, Germany

**Keywords:** medical students, quality of life, nutrition, SF-36, physical performance, endurance capacity

## Abstract

Background: Medical students are exposed to high cognitive demands as well as to a high learning effort, which as a consequence can lead to a limited quality of life (Qol) with reduced physical performance and unhealthy eating behaviors. The aim of this retrospective analysis was to evaluate the abovementioned factors and their relationship to each other. Methods: We included 380 medical students (167 men, 213 women, age 22.2 ± 3.9 yrs) who participated in the sports medicine elective subject. Qol was measured with the SF-36 questionnaire, and endurance capacity was measured by using an incremental running test. Daily dietary intake was measured using a 7-day diary protocol. Depending on sex and the maximum speed achieved, students were divided into three performance groups. Results: Men achieved higher maximal speed, heart rate, and lactate. Carbohydrates and fat intake did not meet recommendations in either group. Dietary fibre intake differed significantly between the performance groups in men and women, with the better groups having higher intakes. Conclusions: Our data do not suggest increased risk or health-damaging behaviors in medical students compared with the general population. Irrespective of this, incentives should be set to enable a healthy life even during complex studies with a high learning effort.

## 1. Introduction

Due to the high mental demands placed on medical students during their studies, quality of life (Qol), health behavior, and stress management are a particular focus for assessing their health status. With regard to the psychological demands and the occurrence of depressive symptoms, possible solutions for dealing with study-specific stressors should be found at an early stage [1]. The percentages of mental health disorders are alarmingly high for stress (55%), depression (30%), anxiety disorders (20%), and burnout (15%), with women partially affected more often than men [2,3].

Regular physical activity is an essential part of a healthy lifestyle and prevents the development of a variety of illnesses such as cardiovascular, metabolic, psychiatric, or oncologic diseases, as well as obesity, falls, cognitive impairments, osteoporosis, and muscular weakness [4,5,6]. However, the minimum of 150 minutes of physical activity per week recommended by the World Health Organization (WHO) for adults [7] is not achieved in a large number of cases. According to a recent status report, 44% of all women and 40% of all men in Germany do not reach the recommendation, and 88% of girls and 80% of boys perform too little daily life physical activity [8]. Regular participation in exercise and frequent physical activities leads to an improvement in physical fitness [9], whereby the level of endurance capacity plays a decisive role in the reduction of cardiovascular mortality [10]. This is all the more crucial since evidence suggests that medical students also have impaired physical performance [11].

Another essential factor for a healthy lifestyle and a higher life expectancy, as well as for the prevention of many chronic diseases, is a healthy and wholesome diet [12]. A Chinese study found unhealthy eating behaviors not in local but in international students with a high intake of fast foods and carbonated drinks [13]. In view of the fact that medical students are supposed to serve as multipliers for a healthy lifestyle, it is even more important that they internalize this behavior already during their medical studies and pass on these guidelines to their patients, especially because many of them turn to their physicians for advice on healthy diets [14].

Considering the high academic stress level, the aim of this retrospective analysis was to evaluate the Qol, physical performance, and eating behaviors of German medical students and their relationship to each other.

## 2. Material and Methods

In the present study, a total of 380 medical students who participated in the sports medicine elective subject from 2010–2019 were examined. As an alternative to this elective, students could also select other topics. Today, there are 14 subjects to choose from such as history of medicine, sports orthopedics, applied anatomy, or experimental pulmonology. Contents of the present elective are, among others, various healthy lifestyle contents and endurance capacity including lactate thresholds. At the end of each three-day seminar, students wrote a test based on their individual results to target possible lifestyle changes (e.g., weight reduction for obesity, dietary changes for low fibre intake, etc.). Based on this test, an individual meeting was held with a physician to discuss the intended changes. Dietary intake and Qol were assessed in the week prior to the seminar.

After consultation with the ethics committee of the Hannover Medical School, further approval was not necessary, since all data were routinely collected during the seminar and evaluated pseudonymously.

## 3. Quality of Life

Qol was assessed using the SF-36 questionnaire that consists of 36 questions and assesses the student’s health status on eight dimensions (physical functioning, role physical, bodily pain, general health, vitality, social functioning, role emotional, and mental health) [15]. The SF-36 questionnaire is scored from 0 to 100 for each dimension. These scores describe the patient’s state of health in the respective dimension, which can then be evaluated using comparison tables [16,17,18]. In addition to these dimensions, a physical and a mental sum score can be calculated.

## 4. Endurance Capacity

A step run test was used to measure endurance capacity, with several 800 m runs at increasing speeds up to individual performance limit. Before the first run, resting heart rate was noted, and resting lactate was taken from the earlobe. The 800 m runs were conducted on a 200 m track, and a one-minute break was taken after each run, during which heart rate was noted and lactate was measured. The initial speed was 2.0 m/s and was increased by 0.5 m/s with each run until exhaustion. The distance should be completed at the prescribed speed, with an acoustic signal for control, where markers had to be reached every 50 m. The maximum speed achieved was noted as endurance capacity (v_max_). If the speed could not be maintained over 800 m, the distance was noted and v_max_ was adjusted in relation to 800 m. One additional lactate sample was taken three minutes after the last run. Depending on v_max_, the students were divided into three performance groups: worst represented the first tercile, middle the second, and best the third tercile. The terciles were calculated separately for men and women.

## 5. Dietary Intake

Food intake was recorded using a 7-day diary protocol. All students were previously given written information on how to complete the protocol. Entries were made on a standardized sheet on which foods and drinks and the quantity consumed were always indicated. Foods and drinks that were not listed were entered as free text. Intakes of energy, macro- and micronutrients, fibre, water, alcohol, and fatty acids were quantitatively analyzed daily. Reference values were taken from the German Nutrition Society (DGE) [19]. The data were analyzed using DGE-PC (Version 5.1.0.048, GOE mbh, Linden, Germany).

## 6. Statistic

All data are given as mean ± standard deviation. Normal distribution was tested using the Kolmogorov-Smirnov test. Differences between men and women were tested with an unpaired *t*-test for parametric data and a Mann-Whitney-U test for non-parametric data with Hedges’ gas effect size. Differences between the three performance groups were computed with an ANOVA with eta squared η^2^ as the effect size. All post hoc tests were corrected after Bonferroni. The significance level was set at 0.05. All calculations were conducted with SPSS (Version 28, Armonk, NY, USA).

## 7. Results

A total of 380 students were included in the analysis, 167 men and 213 women, with a mean age of 22.2 ± 3.9 years. There were no significant differences in age between sexes (s. Table 1). Height, weight, BMI, and waist circumference were higher in men than women. Heart rate and lactate at rest showed no significant difference between gender, but men achieved higher v_max_, maximal heart rate, and maximal lactate than women.

Divided into performance groups, the best group of men had significantly lower weight, BMI, waist and hip circumference, and waist-height ratio than the worst and middle groups (s. Table 2). The worst group of men showed significantly higher resting heart rates than the middle and the best group. The best group of men achieved higher maximal lactate than the two other groups.

In contrast to men, the best group of women only had significantly lower BMI and waist-height ratio than the worst group (Table 3). The best group of women had lower resting heart rates and achieved higher maximal lactate than the two other groups.

**Figure 1:**
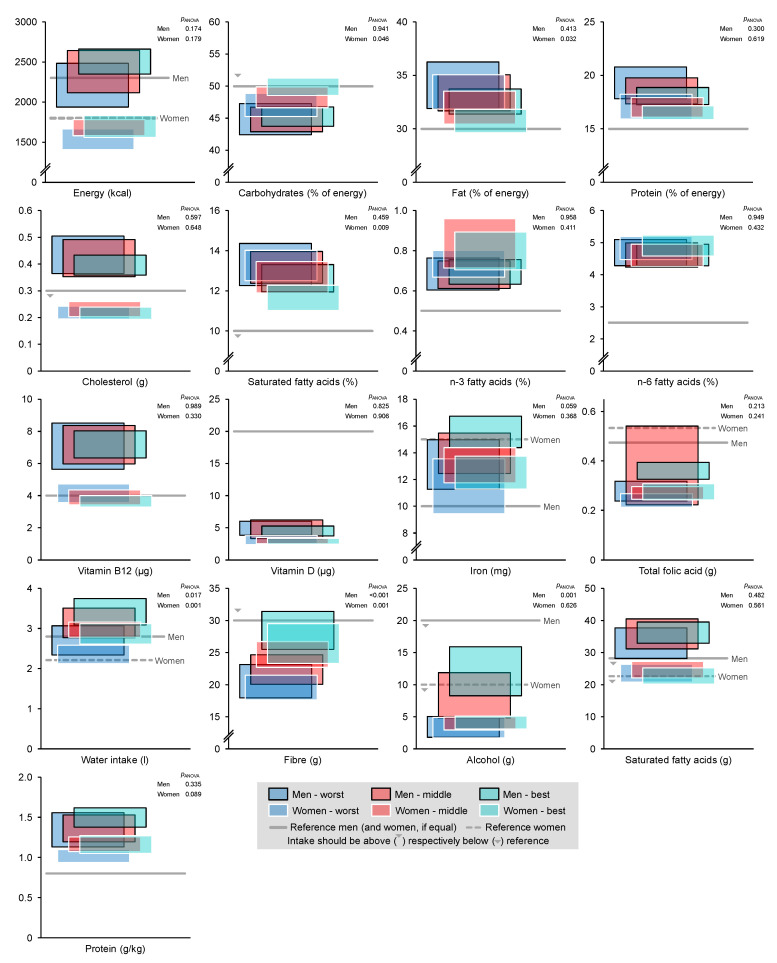
Dietary intake of men (*n* = 167) and women (*n* = 213), divided by performance groups. Displayed are 95% confidence intervals. *p*_ANOVA_: *p*-value of ANOVA between groups divided by sex.

Dietary intakes are displayed in Figure 1. Energy intake showed no differences between performance groups and were in line with the reference values. The distribution of energy among macronutrients differed significantly only in women for carbohydrate and fat, with the best group achieving reference values. Cholesterol intake did not differ between performance groups, and only women achieved the recommendations. The distribution of fatty acids did not reach reference values. The best group of women had a significantly lower distribution of saturated fatty acids than the two other groups. Dietary fibre intake differed significantly between the performance groups in men and women, with the better groups having higher intakes. Dietary iron intake did not differ between the performance groups and was below reference values in both men and women.

**Figure 2:**
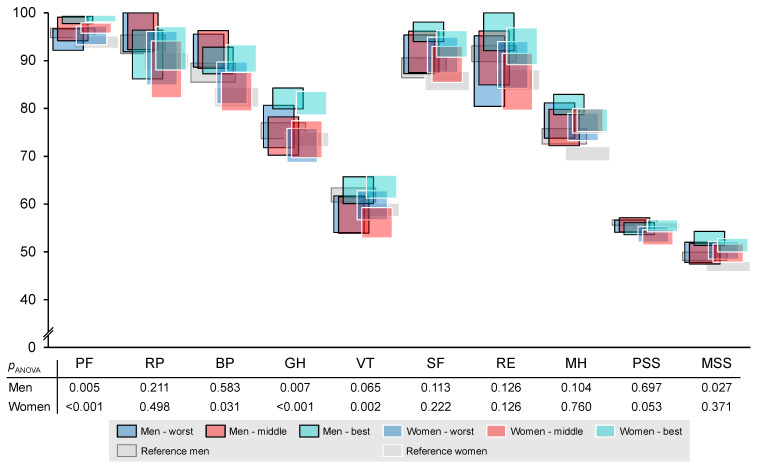
Quality of life of men (*n* = 167) and women (*n* = 213), divided by performance groups. Displayed are 95% confidence intervals. PF: physical functioning; RP: role physical; BP: bodily pain; GH: general health; VT: vitality; SF: social functioning; RE: role emotional; MH: mental health; PSS: physical sum score; MSS: mental sum score; *p*_ANOVA_: *p*-value of ANOVA between groups.

Quality of life is displayed in Figure 2. Men’s quality of life differed significantly between the performance groups in terms of physical functioning (η² = 0.06), general health (η² = 0.06), and mental sum score (η² = 0.04), with the best group reaching higher values. According to the reference values, the best group of men was above the reference values for physical functioning, general health, social functioning, mental health, and mental sum score. Women’s quality of life differed significantly in terms of physical functioning (η² = 0.07), bodily pain (η² = 0.03), general health (η² = 0.07), and vitality (η² = 0.06), with the best group reaching higher values. According to the reference values, the best group of women was above the reference values for physical functioning, bodily pain, general health, vitality, social functioning, and emotional role. All groups of women were above reference values for mental health and mental sum score.

## 8. Discussion

The main finding of our study is that students who participated in the elective sports medicine subject performed surprisingly well in the areas of endurance capacity, Qol, and nutrition. Regardless, it appeared that, in the best performance group, several data related to diet as well as Qol were significantly better.

Several studies report about frequent impairment of medical students by mental health disorders, resulting in reduced academic performance as well as in permanent health impairment [2,20,21]. Often-used positive coping strategies that can counteract the high mental burden are the search for social support in the family, within friends or fellow students, active coping, religion, and sports (2). Negative strategies such as alcohol/substance abuse as well as isolation and distancing (2) should absolutely be prevented and may also be associated with burnout syndrome, depressive disorders, and other psychiatric comorbidities [22,23]. Therefore, it is all the more important to offer strategies against high stress levels in order to avoid manifesting illnesses or to provide adequate therapies to prevent chronification. Overall, in our collective, there was no evidence for the presence of excessive stress or the presence of mental health diseases, and altogether, age-appropriate normal values for Qol were achieved in the SF-36 questionnaire [18]. The fact that some subscales such as physical functioning, general health, social functioning, and mental health were significantly better in the best performance group can be interpreted as an indication that the extent of physical performance has a significant influence on mental as well as physical and social Qol. This was confirmed by Yorks et al. [24], who could find an improvement in regard to perceived stress and also in physical, mental, and emotional components of Qol, where the effects achieved were mainly attributed to exercising in a group.

Nevertheless, should the individual evaluation of a student reveal indications of mental overload, specific measures of course must be undertaken.

Both the level of regular physical activity and the absolute physical capacity are significant predictors of cardiovascular mortality [25,26]. Although the initiation of physical activity is possible at an older age and can lead to an improvement in exercise capacity, the foundation for this is laid during childhood and adolescence [27]. The influence of exercise capacity on cardiovascular mortality can be estimated to be higher than the influence of physical activity [26]. In a Thai study, medical students were shown to have poorer performance on a cycle ergometer in terms of VO_2max_ compared to the normal population, with no difference for sex [11]. In contrast, performers of this elective on average can be characterized by a normal to excellent performance with better values for the males. Additionally, in individual cases of impaired running performance, the cause must be clarified, and if necessary, a specifically adapted training program should be initiated. Stephans et al. found a significant relationship between aerobic fitness and the academic performance measures in relation to pre-clerkship grade point average in medical army students [28]. This could be an indication that a higher physical performance is also associated with an increased willingness to perform in studies.

There is also evidence that physical activity improves Qol and reduces burnout with a dose dependent effect [29]. The authors of this review conclude that physical activity should be integrated into medical studies to improve the well-being of medical students.

To prevent the risk of intoxication or to avoid a deficiency for many nutrients, a daily upper intake level and a minimum daily requirement were defined [30]. The average values for most of the substances (except for fat and carbohydrate intake) examined indicate a predominantly healthy dietary behavior.

Thereby, the best performance group (with the exception of alcohol consumption, which was increased in this group but still below the average recommended by the DGE (20 g/d for men and 10 g/d for women)) had a healthier diet with comparatively lower fat content, lower saturated fat, and increased fibre intake.

In general, however, it can be recommended to all groups to increase fibre content and to limit fat intake. The increased fluid intake is presumably due to the increased physical activity in the better performance groups. Alcohol intake has been part of almost all human cultures for thousands of years and, if handled responsibly on a moderate level, can contribute positively to human health (in particular wine) [31,32] and Qol [33]. On the other hand, a Polish study found an increased rate of alcohol abuse resulting in depression and suicidal ideation in medical students and young doctors; therefore, a regular alcohol intake is not recommended [34].

In summary, the poor knowledge of healthy eating among medical students described in individual studies [35] does not apply to our collective. However, because nutrition plays a crucial role in a healthy lifestyle and currently is insufficiently integrated into medical studies worldwide, recommendations were made to include nutrition education in medical studies [36].

## 9. Limitations

For the overall assessment of our study, some important limitations must be taken into account. First, the subject of sports medicine was only one of several electives that the students of the semester could choose, and we assumed that this subject was mainly selected by those with a high affinity to sports and a healthy lifestyle. Furthermore, the participation took place in the second year of study, where the burden of studying possibly had not yet had such an impact on Qol.

Food intake was recorded during the week before the three-day seminar using a 7-day diary; thus, there may be discrepancies between the currently documented intake situation and the otherwise usual diet.

To some extent, the SF-36 questionnaire may indicate mental abnormalities, but specific questionnaires should be used for screening stress or anxiety disorders.

Exercise was performed as a running test; thus, a comparison with physical capacity achieved on the bicycle ergometer was accordingly difficult. Physical activity besides training is a basic requirement for exercise capacity, and because of the good results in the step run test, we believe that most of the students were physically active to a large extent. Unfortunately, we did not specially ask for daily activities, e.g., with a questionnaire.

## 10. Conclusions

As one of the first studies in this area, we found near-normal Qol scores at our medical students. We attribute this to the high interest in physical activity and healthy lifestyles taught in our elective. We also assume that regular exercise and a good performance are important resilience factors in the context of medical studies, and students should be motivated to remain active even in phases with time shortage resulting from high exam stress. Independently of this, early psychological intervention should take place if there are signs of mental problems in order to prevent permanent damage.

## Figures and Tables

**Table 1:** Characteristics divided by gender.

Parameter	Men		Women		*p*	g
	Mean ± SD	*n*	Mean ± SD	*n*		
Age (years)	22.3 ± 3.6	167	22.2 ± 4.2	213	0.293	0.02
Height (m)	1.81 ± 0.07	167	1.69 ± 0.07	213	<0.001	1.73
Weight (kg)	77.8 ± 10.4	167	61.0 ± 8.3	213	<0.001	1.81
BMI (kg/m²)	23.7 ± 2.7	167	21.3 ± 2.4	213	<0.001	0.94
Hip circumference (cm)	91.4 ± 7.7	165	90.1 ± 8.0	213	0.068	0.17
Waist circumference (cm)	82.7 ± 7.7	165	69.9 ± 5.6	213	<0.001	1.91
Waist-hip ratio	90.7 ± 7.4	165	78.0 ± 6.1	213	<0.001	1.89
Waist-height ratio	45.6 ± 4.4	165	41.4 ± 3.6	213	<0.001	1.07
Heart rate_rest_ (bpm)	90.2 ± 14.1	165	90.6 ± 15.1	213	0.838	−0.02
Lactate_rest_ (mmol/L)	1.06 ± 0.35	154	1.05 ± 0.32	201	0.816	0.01
v_max_ (m/s)	4.55 ± 0.73	167	3.84 ± 0.53	213	<0.001	1.13
Heart rate_max_ (bpm)	200 ± 8	167	195 ± 11	213	<0.001	0.50
Lactate_max_ (mmol/L)	11.65 ± 2.77	167	9.72 ± 2.65	213	<0.001	0.72

BMI: Body mass index; g: effect size Hedge’s g; v: velocity.

**Table 2:** Men’s characteristics, divided by performance groups.

Parameter	Worst		Middle		Best		*p*	η²
	Mean ± SD	*n*	Mean ± SD	*n*	Mean ± SD	*n*		
Age (years)	22.8 ± 4.2	52	22.8 ± 3.4	46	21.5 ± 2.9	69	0.063	0.03
Height (m)	1.80 ± 0.08	52	1.82 ± 0.07	46	1.82 ± 0.07	69	0.166	0.02
Weight (kg)	80.2 ± 12.8 ^b^	52	79.7 ± 9.3 ^c^	46	74.8 ± 8.4 ^b,c^	69	0.007	0.06
BMI (kg/m²)	24.8 ± 3.2 ^b^	52	24.1 ± 2.8 ^c^	46	22.5 ± 1.7 ^b,c^	69	<0.001	0.13
Hip circumference (cm)	93.7 ± 8.4 ^b^	52	92.7 ± 7.7 ^c^	45	88.8 ± 6.4 ^b,c^	68	<0.001	0.08
Waist circumference (cm)	84.9 ± 9.9 ^b^	52	83.7 ± 7.1 ^c^	45	80.2 ± 5.4 ^b,c^	68	0.002	0.07
Waist-hip ratio	90.7 ± 7.4	52	90.6 ± 7.4	45	90.7 ± 7.5	68	0.996	<0.01
Waist-height ratio	47.3 ± 5.3 ^b^	52	46.1 ± 4.5 ^c^	45	44.1 ± 2.9 ^b,c^	68	<0.001	0.10
Heart rate_rest_ (bpm)	98.4 ± 13.2 ^a,b^	51	89.0 ± 11.2 ^a^	45	85.0 ± 13.8 ^b^	69	<0.001	0.16
Lactate_rest_ (mmol/L)	1.12 ± 0.45	43	1.07 ± 0.34	42	1.00 ± 0.27	69	0.205	0.02
v_max_ (m/s)	3.63 ± 0.37 ^a,b^	52	4.35 ± 0.13 ^a,c^	46	5.14 ± 0.32 ^b,c^	69	<0.001	0.82
Heart rate_max_ (bpm)	199 ± 10	52	200 ± 10	46	199 ± 7	69	0.805	<0.01
Lactate_max_ (mmol/L)	10.66 ± 2.90 ^b^	52	11.25 ± 2.42 ^c^	46	12.66 ± 2.58 ^b,c^	69	<0.001	0.10

BMI: Body mass index; v: velocity; η²: effect size eta squared. ^a^ *p* < 0.05 post hoc worst vs. middle; ^b^ *p* < 0.05 post hoc worst vs. best; ^c^ *p* < 0.05 post hoc middle vs. best.

**Table 3:** Women’s characteristics, divided by performance groups.

Parameter	Worst		Middle		Best		*p*_ANOVA_	η²
	Mean ± SD	*n*	Mean ± SD	*n*	Mean ± SD	*n*		
Age (years)	23.8 ± 5.8 ^a,b^	58	21.6 ± 3.0 ^a^	74	21.6 ± 3.4 ^b^	81	0.003	0.05
Height (m)	1.68 ± 0.06	58	1.70 ± 0.07	74	1.70 ± 0.06	81	0.211	0.01
Weight (kg)	62.2 ± 10.9	58	61.2 ± 8.2	74	60.0 ± 5.9	81	0.293	0.01
BMI (kg/m²)	22.0 ± 3.3 ^b^	58	21.2 ± 2.1	74	20.8 ± 1.7 ^b^	81	0.012	0.04
Hip circumference (cm)	91.9 ± 9.4	58	89.6 ± 7.3	74	89.1 ± 7.4	81	0.102	0.02
Waist circumference (cm)	71.2 ± 7.6	58	69.6 ± 5.1	74	69.3 ± 4.2	81	0.115	0.02
Waist-hip ratio	77.8 ± 7.2	58	77.9 ± 4.9	74	78.1 ± 6.3	81	0.940	<0.01
Waist-height ratio	42.5 ± 4.8 ^b^	58	41.1 ± 3.1	74	40.9 ± 2.8 ^b^	81	0.023	0.04
Heart rate_rest_ (bpm)	96.3 ± 14.2 ^b^	58	91.6 ± 14.0 ^c^	74	85.6 ± 15.1 ^b,c^	81	<0.001	0.08
Lactate_rest_ (mmol/L)	1.05 ± 0.39	53	1.07 ± 0.31	72	1.04 ± 0.28	76	0.897	<0.01
v_max_ (m/s)	3.06 ± 0.22 ^a,b^	58	3.60 ± 0.15 ^a,c^	74	4.21 ± 0.34 ^b,c^	81	<0.001	0.77
Heart rate_max_ (bpm)	192 ± 12	58	195 ± 11	74	196 ± 9	81	0.149	0.02
Lactate_max_ (mmol/L)	8.59 ± 2.67 ^b^	58	9.25 ± 2.31 ^c^	74	10.94 ± 2.46 ^b,c^	81	<0.001	0.14

BMI: Body mass index; v: velocity; η²: effect size eta squared. ^a^ *p* < 0.05 post hoc worst vs. middle; ^b^ *p* < 0.05 post hoc worst vs. best; ^c^ *p* < 0.05 post hoc middle vs. best.

## Data Availability

The datasets used and/or analyzed during the current study are available from the corresponding author on reasonable request.

## References

[1] Jurkat H., Höfer S., Richter L., Cramer M., Vetter A. (2011). Lebensqualität, Stressbewältigung Und Gesundheitsförderung Bei Studierenden Der Human-Und Zahnmedizin. DMW-Dtsch. Med. Wochenschr..

[2] Sattar K., Yusoff M.S.B., Arifin W.N., Yasin M.A.M., Nor M.Z.M. (2022). Effective Coping Strategies Utilised by Medical Students for Mental Health Disorders during Undergraduate Medical Education-a Scoping Review. BMC Med. Educ..

[3] Samaranayake C., Fernando A.T. (2011). Satisfaction with life and depression among medical students in Auckland, New Zealand. New Zealand Med. J..

[4] McPhee J.S., French D.P., Jackson D., Nazroo J., Pendleton N., Degens H. (2016). Physical activity in older age: Perspectives for healthy ageing and frailty. Biogerontology.

[5] Mikkelsen K., Stojanovska L., Polenakovic M., Bosevski M., Apostolopoulos V. (2017). Exercise and Mental Health. Maturitas.

[6] Mctiernan A., Friedenreich C.M., Katzmarzyk P.T., Powell K.E., Macko R., Buchner D., Pescatello L.S., Bloodgood B., Tennant B., Vaux-Bjerke A. (2019). Physical Activity in Cancer Prevention and Survival: A Systematic Review. Med. Sci. Sports Exerc..

[7] World Health Organization (2020). WHO Guidelines on Physical Activity and Sedentary Behaviour: Web Annex: Evidence Profiles.

[8] World Health Organization (2022). Global Status Report on Physical Activity.

[9] Chodzko-Zajko W.J., Proctor D.N., Fiatarone Singh M.A., Minson C.T., Nigg C.R., Salem G.J., Skinner J.S., American College of Sports Medicine (2009). American College of Sports Medicine position stand. Exercise and physical activity for older adults. Med. Sci. Sports Exerc..

[10] Warburton D.E.R., Nicol C.W., Bredin S.S.D. (2006). Health benefits of physical activity: The evidence. Can. Med. Assoc. J..

[11] Tongprasert S., Wattanapan P. (2007). Aerobic capacity of fifth-year medical students at Chiang Mai University. J. Med Assoc. Thail..

[12] Li Y., Schoufour J., Wang D.D., Dhana K., Pan A., Liu X., Song M., Liu G., Shin H.J., Sun Q. (2020). Healthy lifestyle and life expectancy free of cancer, cardiovascular disease, and type 2 diabetes: Prospective cohort study. BMJ.

[13] Ul Haq I., Mariyam Z., Li M., Huang X., Jiang P., Zeb F., Wu X., Feng Q., Zhou M. (2018). A Comparative Study of Nutritional Status, Knowledge Attitude and Practices (KAP) and Dietary Intake between International and Chinese Students in Nanjing, China. Int. J. Environ. Res. Public Health.

[14] Snetselaar L., Malville-Shipan K., Ahrens L., Smith K., Chenard C., Stumbo P., Gordon J., Thomas A. (2004). Raising Medical Students’ Awareness of Nutrition and Fitness in Disease Prevention: Nutrition and Fitness Program at the University of Iowa. Med. Educ. Online.

[15] Bullinger M. (1996). Assessment of health related quality of life with the SF-36 Health Survey. Die Rehabil..

[16] Hays R.D., Sherbourne C.D., Mazel R.M. (1993). The rand 36-item health survey 1.0. Health Econ..

[17] Ware J.E. (2000). SF-36 Health Survey Update. Spine.

[18] Ellert U., Kurth B. (2013). Health-Related Quality of Life in Adults in Germany. Bundesgesundheitsbl.

[19] (2022). German Nutrition Society. www.Dge.de/wissenschaft/referenzwerte.

[20] Akhtar M., Herwig B.K., Faize F. (2019). Depression and Anxiety among International Medical Students in Germany: The Predictive Role of Coping Styles. J. Pak. Med Assoc..

[21] Steiner-Hofbauer V., Holzinger A. (2020). How to Cope with the Challenges of Medical Education? Stress, Depression, and Coping in Undergraduate Medical Students. Acad. Psychiatry.

[22] González-Urbieta I., Alfonzo A., Aranda J., Cameron S., Chávez D., Duré N., Pino A., Penner D., Ocampo S., Villalba S. (2020). Burnout Syndrome and Alcohol Dependence in Medical Students. Med. Clínica Soc..

[23] Vaysse B., Gignon M., Zerkly S., Ganry O. (2014). Alcohol, tobacco, cannabis, anxiety and depression among second-year medical students. Identify in order to act. Santé Publique.

[24] Yorks D.M., Frothingham C.A., Schuenke M.D. (2017). Effects of Group Fitness Classes on Stress and Quality of Life of Medical Students. J. Am. Osteopat. Assoc..

[25] Löllgen H., Böckenhoff A., Knapp G. (2009). Physical Activity and all-Cause Mortality: An Updated Meta-Analysis with Different Intensity Categories. Int. J. Sports Med..

[26] Williams P.T. (2001). Physical fitness and activity as separate heart disease risk factors: A meta-analysis. Med. Sci. Sports Exerc..

[27] Fonhus M.S., Brurberg K.G., Kirkehei I., Strom V., Reinar L.M. (2018). Effectiveness of Physical Training Among Children and Adolescents with Habilitation Needs.

[28] Stephens M.B., Dong T., Durning S.J. (2015). Physical Fitness and Academic Performance: A Pilot Investigation in USU Medical Students. Mil. Med..

[29] Taylor C.E., Scott E.J., Owen K. (2022). Physical activity, burnout and quality of life in medical students: A systematic review. Clin. Teach..

[30] Institute of Medicine (US) Panel on Micronutrients (2001). Dietary Reference Intakes for Vitamin A, Vitamin K, Arsenic, Boron, Chromium, Copper, Iodine, Iron, Manganese, Molybdenum, Nickel, Silicon, Vanadium, and Zinc.

[31] Worm N., Belz G., Stein-Hammer C. (2013). Moderate Wine Consumption and Prevention of Coronary Heart Disease. Dtsch. Med. Wochenschr..

[32] Arranz S., Chiva-Blanch G., Valderas-Martínez P., Remon A.M., Raventós R.M.L., Estruch R. (2012). Wine, Beer, Alcohol and Polyphenols on Cardiovascular Disease and Cancer. Nutrients.

[33] Valencia-Martín J.L., Galán I., Guallar-Castillón P., Rodríguez-Artalejo F. (2013). Alcohol drinking patterns and health-related quality of life reported in the Spanish adult population. Prev. Med..

[34] Gajda M., Sedlaczek K., Szemik S., Kowalska M. (2021). Determinants of Alcohol Consumption among Medical Students: Results from POLLEK Cohort Study. Int. J. Environ. Res. Public Health.

[35] Dolatkhah N., Aghamohammadi D., Farshbaf-Khalili A., Hajifaraji M., Hashemian M., Esmaeili S. (2019). Nutrition knowledge and attitude in medical students of Tabriz University of Medical Sciences in 2017–2018. BMC Res. Notes.

[36] Crowley J., Ball L., Hiddink G.J. (2019). Nutrition in medical education: A systematic review. Lancet Planet. Health.

